# Effect of enhanced recovery after surgery protocol for laparoscopic hysterectomy in benign indications: a retrospective cohort study

**DOI:** 10.3389/fmed.2026.1876613

**Published:** 2026-06-29

**Authors:** Yang Liu, Yanping Fang, Xiaolei Zhao

**Affiliations:** Department of Gynecology, The First People’s Hospital of Jiangxia District, Hubei University of Medicine, Wuhan, Hubei, China

**Keywords:** enhanced recovery after surgery, gynecologic surgical procedures, hysterectomy, laparoscopic, perioperative care, treatment outcome

## Abstract

**Objective:**

Enhanced Recovery After Surgery (ERAS) protocols have been proven to optimize recovery in various surgical disciplines, including gynecologic surgery. However, their impact on laparoscopic hysterectomy for benign conditions remains less explored. This study aims to evaluate the effect of the ERAS protocol on perioperative outcomes in patients undergoing laparoscopic hysterectomy for benign gynecologic conditions.

**Methods:**

A retrospective cohort study was conducted at our Hospital between January 2024 and June 2025. Patients undergoing laparoscopic hysterectomy were divided into the ERAS group (*n* = 54) and the conventional management group (*n* = 59) based on perioperative management during hospitalization. The primary outcomes measured were length of hospital stay, time to first ambulation, and time to first flatus. Secondary outcomes included complications, postoperative pain assessed using Visual Analog scale (VAS), gastrointestinal dysfunction assessed using the Simplified Postoperative Nausea and Vomiting Impact Scale (SPONVIS), and patient satisfaction assessed using Patient Satisfaction Questionnaire-18 (PSQ-18).

**Results:**

The ERAS group demonstrated a significant reduction in hospital stay (4.51 ± 1.21 days vs. 6.67 ± 1.08 days, *p* < 0.001), time to first ambulation (17.29 ± 3.69 h vs. 20.61 ± 4.11 h, *p* < 0.001), and time to first flatus (11.05 ± 2.89 h vs. 14.32 ± 2.31 h, *p* < 0.001) compared to the conventional management group. Additionally, the ERAS group showed lower VAS scores, SPONVIS scores, and overall complication rate (all *p* < 0.05).

**Conclusion:**

The implementation of the ERAS protocol in laparoscopic hysterectomy for benign conditions may improve perioperative outcomes, including reducing hospital stay, accelerating recovery, and enhancing patient satisfaction without increasing complication rates.

## Introduction

Laparoscopic hysterectomy is widely used for benign gynecologic conditions because it is associated with less blood loss, shorter hospitalization, and faster recovery than open surgery (1, 2). Nevertheless, postoperative recovery after minimally invasive hysterectomy is still influenced by perioperative practices, including fasting, bowel preparation, analgesia, nutrition, catheter management, and mobilization (3, 4).

Enhanced Recovery After Surgery (ERAS) is a multidisciplinary, evidence-based perioperative pathway designed to reduce surgical stress and promote earlier functional recovery (4, 5). In gynecologic surgery, recent reviews and clinical studies suggest that ERAS pathways can shorten hospital stay, reduce pain and postoperative nausea and vomiting, and improve patient experience without increasing readmission or major complications (6–8). However, most available evidence in gynecology has been derived from oncologic surgery or mixed surgical populations, and ERAS implementation varies substantially across institutions (9–11).

Data focusing specifically on laparoscopic hysterectomy for benign indications remain comparatively limited, particularly in routine single-center practice (7, 12). Therefore, this retrospective cohort study evaluated whether implementation of an ERAS protocol was associated with improved perioperative outcomes among patients undergoing laparoscopic hysterectomy for benign gynecologic conditions.

## Materials and methods

### Study design and participants

A retrospective cohort study was conducted at the Hospital between January 2024 and June 2025. During the study period, the ERAS protocol was introduced as a department-led perioperative pathway after discussion among gynecologic surgeons, anesthesiologists, and nursing staff. The implementation was motivated by evidence supporting standardized perioperative care, multimodal analgesia, early oral intake, early mobilization, and faster functional recovery after gynecologic surgery. Group allocation reflected the clinical pathway in use for each patient. The conventional management group (*n* = 59) received standard perioperative care, while the ERAS group (*n* = 54) received enhanced recovery after surgery protocol. The study protocol was approved by the hospital’s Institutional Review Board.

Patients were included if they met the following criteria: age 18–65 years; benign gynecological diagnosis including uterine fibroids, adenomyosis, or dysfunctional uterine bleeding; scheduled for elective laparoscopic hysterectomy (total hysterectomy or subtotal hysterectomy); American Society of Anesthesiologists (ASA) physical status classification I–II; complete medical records available for analysis. Exclusion criteria included: malignant tumor or suspected malignancy; emergency surgery; intraoperative conversion to laparotomy; severe comorbidities including significant cardiac, pulmonary, hepatic, or renal insufficiency; preoperative chronic use of analgesics or corticosteroids; cognitive dysfunction or psychiatric disorders.

### Management approach

All surgical procedures were performed by the same team of experienced gynecological surgeons. Standardized laparoscopic equipment was used for all cases. General anesthesia was administered using a standardized protocol with endotracheal intubation. Intraoperative monitoring included continuous electrocardiography, pulse oximetry, non-invasive blood pressure measurement, end-tidal carbon dioxide monitoring, and body temperature maintenance.

#### Conventional management group

The conventional management group received standard perioperative care according to traditional hospital protocols. Pre-operatively, patients fasted from solid food for at least 8 h and cleared fluids for at least 6 h before surgery. Mechanical bowel preparation with oral laxatives was routinely administered the evening before surgery. No preoperative carbohydrate loading was provided. Preoperative counseling was limited to routine surgical consent and brief verbal instructions regarding the procedure. Intraoperatively, nasogastric tubes were routinely placed after induction of anesthesia and maintained until bowel function returned. Standard fluid management followed traditional protocols with crystalloid infusion. Body temperature maintenance relied on passive warming measures. Standard anesthetic techniques were employed without specific multimodal analgesia protocols. Postoperatively, patients remained fasting until passage of flatus or return of bowel sounds, typically allowing clear liquids on postoperative day 1 and regular diet on day 2. Urinary catheters were maintained for at least 24–48 h postoperatively. Early mobilization was encouraged but not systematically implemented. Pain management consisted primarily of opioid analgesics administered as needed. Antibiotic prophylaxis and thromboprophylaxis followed standard institutional protocols. Discharge criteria included tolerance of regular diet, adequate pain control with oral medications, absence of complications, and patient’s subjective readiness for discharge.

#### ERAS group

The ERAS group received perioperative care based on established ERAS Society guidelines for gynecological surgery, incorporating evidence-based interventions across the preoperative, intraoperative, and postoperative phases. After the surgical decision was made and ERAS eligibility was confirmed, patients received comprehensive education and psychological counseling using standardized educational materials. Patients received detailed information regarding the surgical procedure, expected recovery timeline, pain management strategies, and specific postoperative milestones. Realistic expectations were established, emphasizing active patient participation in the recovery process. Mechanical bowel preparation was omitted unless specifically indicated. Fasting protocols were optimized: patients consumed solid food until 6 h before surgery and were encouraged to drink carbohydrate-rich beverages (12.5% carbohydrate solution, 400 mL) until 2 h preoperatively. Nasogastric tube placement was avoided unless clinically necessary. Prophylactic antibiotic administration followed standard guidelines, with appropriate agents administered within 60 min before surgical incision. Thromboprophylaxis was initiated according to individual risk assessment.

A standardized anesthetic protocol was implemented with emphasis on short-acting agents and rapid recovery. Goal-directed fluid therapy was employed, targeting euvolemia and avoiding fluid overload through minimally invasive hemodynamic monitoring when available. Crystalloid infusion rates were maintained at 3–5 mL/kg/h with judicious use of colloids when indicated. Active body temperature maintenance was achieved through multiple interventions: forced-air warming blankets, warmed intravenous fluids maintained at 37 °C, and increased operating room temperature (21–23 °C) to maintain core body temperature above 36 °C throughout the procedure. Multimodal analgesia strategies were implemented to minimize opioid requirements. This included local anesthetic infiltration at trocar sites (0.25% bupivacaine, 20–30 mL total), intravenous paracetamol (1 g), and non-steroidal anti-inflammatory drugs when not contraindicated. Prophylactic antiemetic medications, including dexamethasone (8 mg) and ondansetron (4 mg), were administered to prevent postoperative nausea and vomiting.

Early oral intake was encouraged, with clear liquids offered 2–4 h postoperatively and advancement to regular diet within 6 h as tolerated. Patients were provided with sugar-free chewing gum beginning 2 h postoperatively, with instructions to chew for 5–10 min three times daily to stimulate bowel function. Urinary catheters were removed within 24 h postoperatively for uncomplicated cases. Early mobilization protocols were systematically implemented: patients were assisted to ambulate on the day of surgery or the following morning, with structured mobilization goals established. Patients were encouraged to sit out of bed and walk in the corridor multiple times daily with progressive increase in activity. Multimodal analgesia was continued postoperatively, combining regular non-opioid analgesics including paracetamol (1 g every 6 h) and non-steroidal anti-inflammatory drugs. Opioid analgesics were reserved for rescue analgesia when pain scores exceeded 4/10 on the VAS despite non-opioid medications.

Daily recovery assessments were conducted using standardized checklists. Discharge criteria were applied systematically: adequate pain control with oral analgesics (pain score ≤4/10), tolerance of solid food without nausea or vomiting, independent mobilization, passage of flatus, absence of complications requiring inpatient management, and patient willingness to be discharged with appropriate home support.

### Outcome measures and data collection

#### Primary outcomes

Length of hospital stay was measured in days from surgery completion to hospital discharge, recorded to the nearest 0.5 day. Time to first ambulation was recorded in hours from surgery completion to first out-of-bed mobilization with or without assistance. Time to first flatus was documented in hours from surgery completion to patient-reported passage of flatus, verified through nursing documentation.

#### Secondary outcomes

(1) Postoperative pain intensity was assessed using the VAS. The VAS is a widely used pain assessment tool, consisting of a 10-cm horizontal line with verbal anchors at each end, with 0 indicating “no pain” and 10 indicating “worst imaginable pain.” Recent validation evidence has supported the consistency of VAS pain scoring in adult populations (13). Pain assessments were conducted at rest at 6, 12, and 24 h postoperatively by ward nurses.

(2) Postoperative nausea and vomiting (PONV) was evaluated at 24 h postoperatively and before discharge using SPONVIS (14). The SPONVIS assesses two dimensions: the impact of nausea on postoperative recovery, scored from 0 (no nausea) to 5 (extreme impact), and the number of vomiting episodes, scored as 0 for no vomiting, 1 for one episode, 2 for two episodes, and 3 for three or more episodes. The total score ranges from 0 to 8, with higher scores indicating greater PONV severity. A score ≥5 is defined as clinically important PONV. The scale has demonstrated excellent reliability (test–retest reliability coefficient 0.95) and responsiveness (effect size 0.78) in postoperative populations (15).

(3) Postoperative complications were documented and classified according to severity. The overall complication rate was calculated as the proportion of patients experiencing at least one complication during the hospitalization or within 30 days postoperatively. Specific complication categories included: infectious complications (fever defined as body temperature >38 °C lasting >24 h, surgical site infection classified according to Centers for Disease Control criteria, urinary tract infection confirmed by positive urine culture with associated symptoms); bleeding complications (postoperative hemorrhage requiring blood transfusion or surgical intervention); urinary system complications (urinary retention requiring catheter reinsertion, bladder injury); and other complications (intestinal obstruction, wound dehiscence, venous thromboembolism). Urinary catheter duration was also included for analysis. Hospital readmission within 30 days postoperatively was recorded, with documentation of the specific reason for readmission and classification as surgery-related or unrelated to the index procedure.

(4) Patient satisfaction was assessed using PSQ-18 administered before hospital discharge. The PSQ-18 has been validated across multiple healthcare settings and populations, demonstrating good psychometric properties with internal consistency coefficients (Cronbach’s *α*) typically ranging from 0.74 to 0.95 and test–retest reliability coefficients from 0.74 to 0.92 (16). The questionnaire evaluates multiple dimensions of care including satisfaction with preoperative education, pain management, nursing care, physician communication, and overall hospital experience.

### Data collection and quality control

Medical records were retrospectively reviewed by trained research coordinators using standardized data collection forms. All data were extracted from the hospital’s electronic medical record system. Data quality control measures included double data entry by independent coordinators and regular audits to ensure accuracy and completeness.

### Statistical analysis

Data normality was assessed using the Shapiro–Wilk test. Continuous variables were presented as mean ± standard deviation for normally distributed data or median (interquartile range) for non-normally distributed data. Categorical variables were presented as frequencies and percentages. For between-group comparisons, independent samples *t*-tests were used for normally distributed continuous variables, while Mann–Whitney *U* tests were employed for non-normally distributed data. Chi-square tests were used for categorical variables with expected frequencies ≥5 in all cells; Fisher’s exact test was employed when expected cell frequency was <5 in any cell. For pain scores measured at multiple time points, comparisons between groups at each time point were performed using Mann–Whitney *U* tests, with Bonferroni correction applied to adjust for multiple comparisons.

All statistical tests were two-sided with significance set at *p* < 0.05 unless otherwise specified. Statistical analysis was performed using SPSS version 26.0 software (IBM Corp., Armonk, NY, United States) or R version 4.0.

## Results

### Baseline characteristics

This analysis included 113 patients, comprising 59 in the conventional management group and 54 in the ERAS group (Table 1). Baseline characteristics across all measured parameters were comparable between groups. Age and body mass index (BMI) distributions were similar. ASA classification, distribution of comorbidities, proportion of prior abdominal surgeries, and perioperative characteristics were equivalent in both groups (all *p* > 0.05).

**Table 1:** Baseline characteristics of two groups.

Characteristics	Conventional management group (*n* = 59)	ERAS group (*n* = 54)	*t*/*χ*^2^	*P*
Demographics				
Age (years)	46.32 ± 8.54	45.78 ± 9.12	0.331	0.741
BMI (kg/m^2^)	23.47 ± 3.21	23.89 ± 3.05	0.728	0.468
ASA classification, *n* (%)			0.142	0.706
Class I	37 (62.71)	32 (59.26)		
Class II	22 (37.29)	22 (40.74)		
Medical comorbidities, *n* (%)				
Hypertension	12 (20.34)	10 (18.52)	0.061	0.805
Diabetes mellitus	7 (11.86)	5 (9.26)	0.214	0.644
Cardiovascular disease	5 (8.47)	4 (7.41)	0.047	0.828
Previous abdominal surgery, *n* (%)	14 (23.73)	11 (20.37)	0.194	0.660
Surgical indication, *n* (%)			1.287	0.732
Uterine fibroids	28 (47.46)	27 (50.00)		
Adenomyosis	18 (30.51)	14 (25.93)		
Dysfunctional uterine bleeding	10 (16.95)	11 (20.37)		
Others	3 (5.08)	2 (3.70)		
Type of hysterectomy, *n* (%)			0.523	0.770
Total laparoscopic hysterectomy	42 (71.19)	37 (68.52)		
Laparoscopic-assisted vaginal hysterectomy	11 (18.64)	12 (22.22)		
Laparoscopic supracervical hysterectomy	6 (10.17)	5 (9.26)		
Perioperative indicators				
Operative time (min)	87.45 ± 18.32	85.67 ± 19.74	0.514	0.608
Estimated blood loss (mL)	112.34 ± 45.67	108.52 ± 42.89	0.469	0.640
Intraoperative fluid administration (mL)	1234.58 ± 287.45	1198.76 ± 302.34	0.660	0.510

Data are presented as mean ± standard deviation or *n* (%). BMI, body mass index; ASA, American Society of Anesthesiologists; ERAS, enhanced recovery after surgery.

Surgical indications were similarly distributed (*p* > 0.05), with uterine fibroids being the most common indication (47.46% vs. 50.00%), followed by adenomyosis (30.51% vs. 25.93%) and dysfunctional uterine bleeding (16.95% vs. 20.37%). There was no significant difference in the type of hysterectomy performed between the two groups (*p* > 0.05), with total laparoscopic hysterectomy being the predominant approach (71.19% vs. 68.52%).

### Primary outcomes

Table 2 presents the comparison of primary outcomes between the two groups. The ERAS group demonstrated significantly shorter length of hospital stay compared to the conventional management group (4.51 ± 1.21 days vs. 6.67 ± 1.08 days, *p* < 0.001). And time to first ambulation was significantly reduced in the ERAS group (17.29 ± 3.69 h) compared to the conventional management group (20.61 ± 4.11 h, *p* < 0.001). Similarly, time to first flatus was significantly shorter in the ERAS group (11.05 ± 2.89 h) compared to the conventional management group (14.32 ± 2.31 h, *p* < 0.001).

**Table 2:** Comparison of primary outcomes.

Indicator	Conventional management group (*n* = 59)	ERAS group (*n* = 54)	*t*	*P*
Length of hospital stay (days)	6.67 ± 1.08	4.51 ± 1.21	10.030	<0.001
Time to first ambulation (hours)	20.61 ± 4.11	17.29 ± 3.69	4.503	<0.001
Time to first flatus (hours)	14.32 ± 2.31	11.05 ± 2.89	6.670	<0.001

Data are presented as mean ± standard deviation. ERAS, enhanced recovery after surgery.

### Secondary outcomes

Table 3 presents the comparison of complications and urinary catheter duration between the two groups. Urinary catheter duration was shorter in the ERAS group (17.67 ± 4.26 h vs. 42.75 ± 9.34 h, *p* < 0.001). The 30-day readmission rate was 5.08% in the conventional management group and 1.85% in the ERAS group (*p* > 0.05), with no statistically significant difference between the two groups. Overall complication rates differed significantly between groups (25.42% vs. 11.11%, *p* = 0.010).

**Table 3:** Comparison of complications and urinary catheter duration between two groups.

Indicator	Conventional management group (*n* = 59)	ERAS group (*n* = 54)	*t*/*χ*^2^	*P*
Urinary catheter duration (hours)	42.75 ± 9.34	17.67 ± 4.26	17.927	<0.001
30-day readmission rate	3 (5.08)	1 (1.85)	1.242	0.265
Overall complication rate	15 (25.42)	6 (11.11)	6.630	0.010
Fever	8 (13.56)	3 (5.56)	2.057	0.151
Surgical site infection	3 (5.08)	1 (1.85)	–	0.620*
Urinary tract infection	2 (3.39)	1 (1.85)	–	1.000*
Postoperative bleeding	1 (1.69)	1 (1.85)	–	1.000*
Intestinal obstruction	1 (1.69)	0 (0.00)	–	1.000*

Categorical data are presented as *n* (%). Continuous data are presented as mean ± standard deviation. ERAS, enhanced recovery after surgery. (−) indicates using Fisher’s exact test.

**Figure 1:**
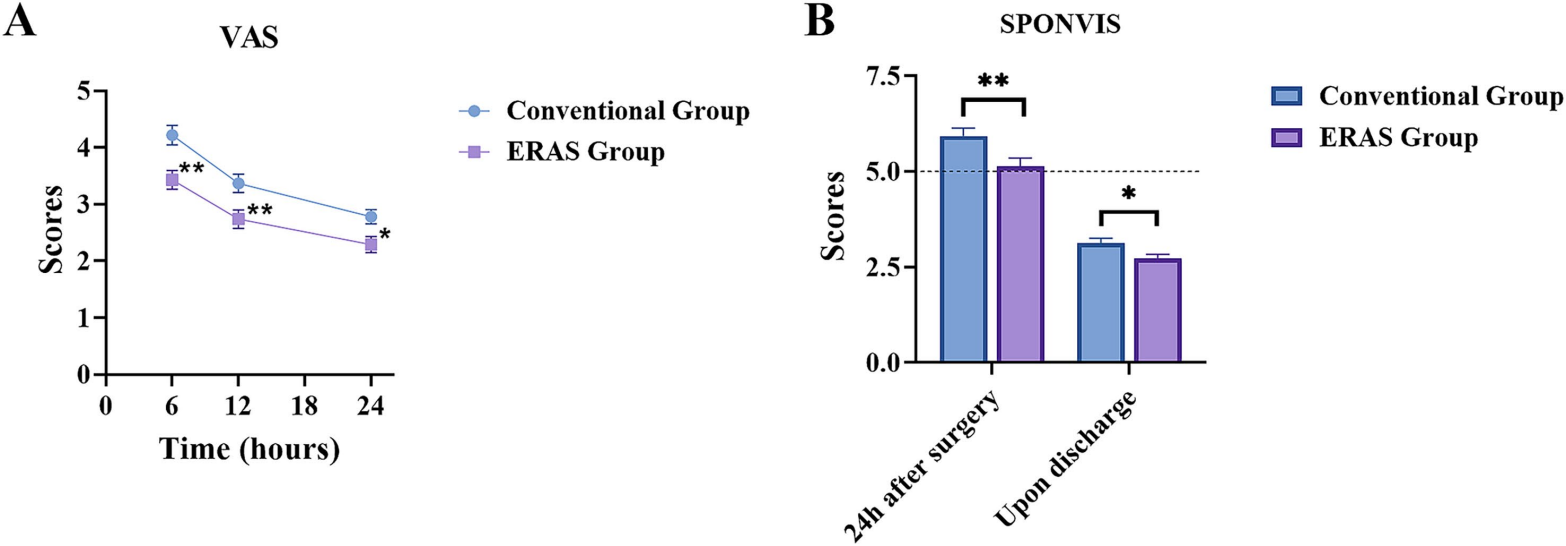
Changes in VAS and SPONVIS scores between two groups. **(A)** Changes in Visual Analog Scale (VAS) at 6, 12, and 24 h postoperatively for the two groups. **(B)** Comparison of Simplified Postoperative Nausea and Vomiting Impact Scale (SPONVIS) Scores between two groups at 24 h after surgery and upon discharge. The dashed line indicates clinically significant PONV when SPONVIS ≥5 points. Compared with the conventional management group at the same time, *p < 0.05, **p < 0.01.

Figure 1A illustrates the changes in VAS scores at three postoperative time points. At 6 h postoperatively, the ERAS group exhibited significantly lower pain scores compared to the conventional management group (*p* < 0.01). This difference persisted at 12 h (*p* < 0.05) and 24 h postoperatively (*p* < 0.05).

As shown in Figure 1B, SPONVIS scores differed significantly between groups at both assessment points. At 24 h postoperatively, the ERAS group demonstrated lower SPONVIS scores compared to the conventional management group (*p* < 0.01), indicating reduced severity of postoperative nausea and vomiting. At discharge, the ERAS group maintained significantly lower SPONVIS scores (*p* < 0.05).

**Figure 2:**
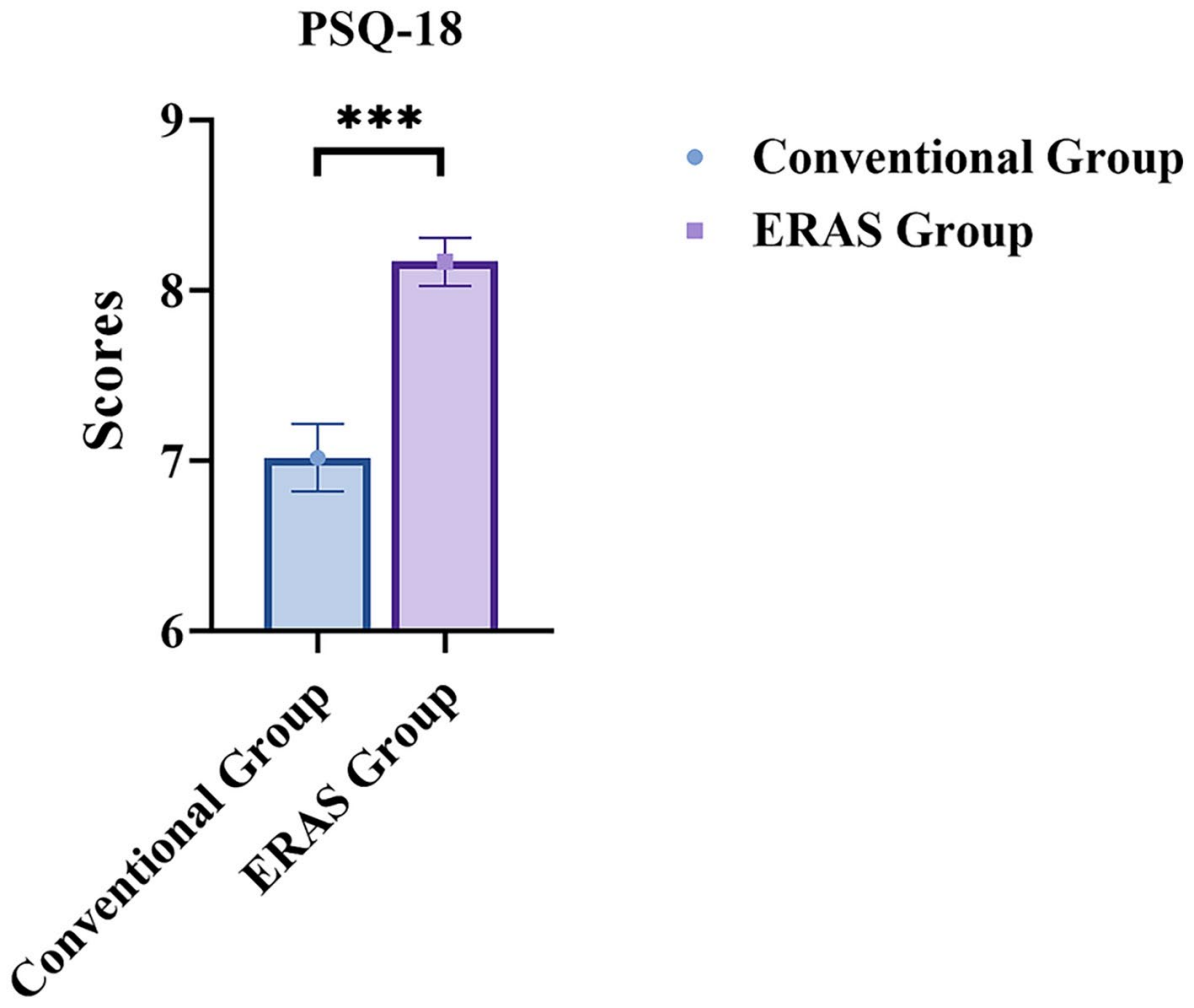
Comparison of patient satisfaction between two groups. Comparison of Patient Satisfaction Questionnaire-18 (PSQ-18) scores between the two groups at discharge. ***p < 0.001.

Figure 2 showed a comparison of PSQ-18 scores at discharge between the two patient groups and demonstrated that the scores in the ERAS group were significantly higher than those in the conventional management group (*p* < 0.001).

## Discussion

This study demonstrated that implementation of an ERAS protocol in laparoscopic hysterectomy for benign conditions was associated with improved perioperative outcomes. Compared with conventional management, the ERAS pathway reduced length of hospital stay by 2.16 days, shortened time to first ambulation and first flatus, lowered postoperative pain and PONV scores, and improved patient satisfaction without increasing 30-day readmission. These findings suggest that ERAS can be feasibly applied to benign-indication laparoscopic hysterectomy in routine clinical practice.

Our findings are consistent with recent systematic reviews and meta-analyses showing that ERAS pathways can reduce length of hospital stay and improve postoperative recovery after gynecologic surgery and hysterectomy (6, 7, 17). The 2.16-day reduction observed in our cohort is clinically meaningful and comparable with recent ERAS studies in hysterectomy and minimally invasive gynecologic surgery (7, 8). Similar benefits have also been reported in studies of minimally invasive or robotic hysterectomy, including improved pain control, earlier gastrointestinal recovery, and reduced postoperative nausea and vomiting (8, 18, 19). However, many previous studies were derived from gynecologic oncology populations, mixed gynecologic procedures, or relatively controlled trial settings. The present study differs by focusing specifically on laparoscopic hysterectomy for benign indications in a routine single-center departmental implementation setting. In addition, we assessed both clinical recovery indicators and patient-centered outcomes, including serial VAS pain scores, SPONVIS-assessed nausea and vomiting, PSQ-18 satisfaction, urinary catheter duration, complications, and 30-day readmission.

The improvements observed may be explained by the combined effects of multiple ERAS components. Early mobilization enhances pulmonary function, promotes gastrointestinal motility, and facilitates earlier discharge (20). In addition, we observed significantly lower pain scores in the ERAS group at three time points postoperatively. This multimodal approach, combining local anesthetic infiltration, acetaminophen, and non-steroidal anti-inflammatory drugs, minimizes opioid-related adverse effects including nausea, vomiting, and ileus (4, 18).

Early oral nutrition represents a paradigm shift from traditional fasting practices. In our protocol, patients received clear liquids within 2–4 h and advanced to regular diet within 6 h postoperatively. The gastrointestinal tract maintains absorptive capacity immediately after minimally invasive surgery, and early feeding reduces insulin resistance and accelerates functional recovery (21). We found the ERAS group demonstrated significantly lower gastrointestinal dysfunction scores and faster time to first flatus, supporting the safety and efficacy of early feeding protocols. Preoperative patient education enhanced engagement in the recovery process. Comprehensive counseling regarding surgical procedures, recovery timelines, and postoperative goals established realistic expectations and improved adherence to protocols. The significantly higher patient satisfaction scores in the ERAS group reflect the importance of patient-centered care in perioperative management. Consistent with multiple systematic review reports, we observed a significant reduction in complication rates under ERAS protocols. This reduction may also result from optimized fluid management, maintenance of normal body temperature, prophylactic use of antiemetic medications, and avoidance of routine nasogastric tube placement (22, 23).

The implementation involved several challenges. Establishing multidisciplinary coordination among surgeons, anesthesiologists, and nursing staff required regular meetings and clear communication. Initial resistance to change from team members accustomed to traditional practices required education regarding evidence supporting ERAS interventions and demonstration of early positive outcomes (10). Patient compliance with early mobilization and oral intake required dedicated nursing support and comprehensive preoperative counseling. However, the cost-effectiveness has been well established, with substantial cost savings through reduced hospital stays offsetting implementation costs (24).

Several limitations of this study should be acknowledged. The retrospective design restricted causal inference and potentially introduced selection bias, despite balanced baseline characteristics between groups. The single-center study design limited the generalizability of findings, and the sample size could be expanded further. Additionally, long-term outcomes beyond 30 days were not examined due to constraints such as medical record availability. Future research will focus on prospective randomized controlled trials with larger sample sizes and multicenter studies to enhance generalizability. Long-term outcome evaluation including quality of life, functional status, and patient-reported outcomes over extended follow-up periods remains a priority (25). Investigation of optimal component combinations and patient-specific factors could enable more individualized perioperative care pathways. Additionally, integration of digital health applications for patient education and remote monitoring may further optimize outcomes (26, 27).

## Conclusion

Implementation of an ERAS protocol for laparoscopic hysterectomy in benign conditions is feasible, safe, and associated with significant improvements in perioperative outcomes. The reductions in hospital stay, faster recovery, improved pain control, and higher patient satisfaction without increases in complications support broader adoption of ERAS principles in benign gynecologic surgery. Successful implementation requires multidisciplinary collaboration, comprehensive patient education, and commitment to evidence-based practice.

## Data Availability

The original contributions presented in the study are included in the article/supplementary material, further inquiries can be directed to the corresponding author.
